# DESIGNING TECHNOLOGY TO SUPPORT HEALTHCARE FOR AGING ADULTS

**DOI:** 10.1093/geroni/igz038.122

**Published:** 2019-11-08

**Authors:** Neil H Charness, Scott R Beach

**Affiliations:** 1 Florida State University, Tallahassee, Florida, United States; 2 University of Pittsburgh, Pittsburgh, Pennsylvania, United States

## Abstract

Healthcare costs are rising in industrialized countries, partly as a function of managing costly chronic care conditions associated with aging populations. Of roughly 3 trillion USD expended in the U.S., almost 90% is spent on those with chronic conditions. Technology is touted as one tool to manage healthcare efficiently. However, human factors research has shown that technological systems that do not take human capabilities into account will fail to be adopted, or if adopted, will be abandoned by users. The Center for Research and Education on Aging and Technology Enhancement (CREATE) will describe research findings for four different facets of healthcare technology. Sara Czaja will provide an overview, describing technology for healthcare support. Caregiver needs are projected to rise rapidly, in part due to aging of the baby boom cohorts. We need new solutions for future generations of older adults as there will be insufficient numbers of caregivers to care for the increased number of older adults given changes in social structures. Wendy Rogers will discuss research on the design and use of televideo and robots to assist with healthcare. Neil Charness will discuss home monitoring technology, particularly practical issues around design, deployment, and maintenance, drawing on studies of heart failure patients and older adult controls. Walter Boot will discuss how gamification of healthcare interventions can help to address the adherence problem for behavior change. Scott Beach, Associate Director & Director of Survey Research Program, University Center for Social and Urban Research, University of Pittsburgh, will serve as discussant.

